# Development of Nanobodies Against Hemorrhagic and Myotoxic Components of *Bothrops atrox* Snake Venom

**DOI:** 10.3389/fimmu.2020.00655

**Published:** 2020-05-07

**Authors:** Henri Bailon Calderon, Verónica Olga Yaniro Coronel, Omar Alberto Cáceres Rey, Elizabeth Gaby Colque Alave, Walter Jhon Leiva Duran, Carlos Padilla Rojas, Harrison Montejo Arevalo, David García Neyra, Marco Galarza Pérez, César Bonilla, Benigno Tintaya, Giulia Ricciardi, Natalia Smiejkowska, Ema Romão, Cécile Vincke, Juan Lévano, Mary Celys, Bruno Lomonte, Serge Muyldermans

**Affiliations:** ^1^Laboratorio de Referencia Nacional de Biotecnología y Biología Molecular, Centro Nacional de Salud Pública, Instituto Nacional de Salud, Lima, Peru; ^2^Laboratorio de Biología Molecular, Universidad Nacional Mayor de San Marcos, Lima, Peru; ^3^Centro Nacional de Producción de Biológicos (INS), Lima, Peru; ^4^Cellular and Molecular Immunology, Vrije Universiteit Brussel, Brussels, Belgium; ^5^Instituto Clodomiro Picado, Facultad de Microbiología, Universidad de Costa Rica, San Jose, Costa Rica

**Keywords:** nanobodies, snake, venom, myotoxic, hemorrhagic, neutralization, *Bothrops atrox*, viperidae

## Abstract

Snake envenoming is a globally neglected public health problem. Antivenoms produced using animal hyperimmune plasma remain the standard therapy for snakebites. Although effective against systemic effects, conventional antivenoms have limited efficacy against local tissue damage. In addition, potential hypersensitivity reactions, high costs for animal maintenance, and difficulties in obtaining batch-to-batch homogeneity are some of the factors that have motivated the search for innovative and improved therapeutic products against such envenoming. In this study, we have developed a set of nanobodies (recombinant single-domain antigen-binding fragments from camelid heavy chain-only antibodies) against *Bothrops atrox* snake venom hemorrhagic and myotoxic components. An immune library was constructed after immunizing a *Lama glama* with whole venom of *B. atrox*, from which nanobodies were selected by phage display using partially purified hemorrhagic and myotoxic proteins. Biopanning selections retrieved 18 and eight different nanobodies against the hemorrhagic and the myotoxic proteins, respectively. *In vivo* assays in mice showed that five nanobodies inhibited the hemorrhagic activity of the proteins; three neutralized the hemorrhagic activity of whole *B. atrox* venom, while four nanobodies inhibited the myotoxic protein. A mixture of the anti-hemorrhagic and anti-myotoxic nanobodies neutralized the local tissue hemorrhage and myonecrosis induced by the whole venom, although the nanobody mixture failed to prevent the venom lethality. Nevertheless, our results demonstrate the efficacy and usefulness of these nanobodies to neutralize important pathologies of the venom, highlighting their potential as innovative therapeutic agents against envenoming by *B. atrox*, a viperid species causing many casualties in South America.

## Introduction

Snakebite envenoming is an important public health problem worldwide, especially in tropical and subtropical countries. Around the world, 5 million snakebites occur each year, affecting mainly the rural populations and causing an estimated 100,000 deaths (1). Victims that survive snake envenoming may suffer from permanent physical sequelae, such as amputation of the affected body parts, as well as psychological sequela, such as depression, which can reduce their productive capacities and affect normal life. Moreover, most snake envenoming accidents in developing countries occur in remote areas, often far away from health services, or where antivenoms are unavailable, which decreases the chances for effective treatment and lifesaving of the victims.

Venomous snakes around the world include the families Viperidae, Elapidae, Atractaspididae, and Colubridae (2), the former two being the most medically relevant. Within the Viperidae family, the genus *Bothrops* is responsible for the most snake envenoming in Central and South America, causing high morbidity and mortality (3).

Antivenom administration is the only effective treatment for snake envenoming. Currently, antivenom production is based on the immunization of equines or ovine with snake venoms according to the species responsible for the accidents in a region or country.

Hyperimmune plasma is obtained and used for purification of whole IgG antibodies (150 kDa) or for obtaining antibody fragments, such as F(ab’)_2_ (100 kDa) or Fab (50 kDa), for the antivenom formulation (4).

Besides conventional antibodies, camelids and some shark species produce naturally a unique type of antibody that is composed of heavy chains only, referred to as heavy-chain-only antibodies (HCAbs) (5). The antigen recognition of these functional HCAbs is comprised in the variable region of their heavy chain [abbreviated as VHH and referred to as Nanobody (Nb)].

Nanobodys are small proteins of approximately 15 kDa; they are the smallest intact antigen-binding fragment (6) that retains the specificity and affinity of the original HCAb in recognizing the antigen (7, 8). The Nbs have promising potential as therapeutic (9–11) and diagnostic tools (12). Their third antigen-binding loop, or CDR3 (complementarity determining region), is longer than that of VH domains of conventional antibodies, and this prolonged loop interacts preferentially with cavities or concave surfaces, such as the active site of enzymes (13). While large clefts or grooves on the surface of antigens are less likely to interact with the flat surface of the paratope of classical antibodies, they are frequently observed to associate with the convex paratope formed mainly by the CDR3 of camelid VHHs (14). Furthermore, the substitution of conserved large and hydrophobic amino acids in the framework-2 region of VH with smaller and hydrophilic amino acids, prevents the Nbs from associating with a VL domain like in classical antibodies. It also renders the isolated Nb soluble in aqueous solutions without any sign of aggregation (8). In addition, Nbs resist exposure to elevated temperatures (15), and they are expressed to high levels in microorganisms, such as *E. coli*, thereby reducing the production cost.

*Bothrops atrox* snake venom has been characterized in previous studies (16) including proteomic analysis, which have determined the predominant presence of metalloproteinases of the SVMP-I and SVMP-III classes in addition to other protein types, such as phospholipase A2 (PLA_2_) and PLA_2_-like homologs, serine proteinases, and disintegrins, among others (17).

SVMPs are relevant toxins of *Bothrops* spp. venoms since many display a potent hemorrhagic effect, especially those of the SVMP-III class (18, 19). On the other hand, basic PLA_2_ and PLA_2_-like proteins induce a strong myotoxic effect leading to local necrosis (20, 21). Together, these two types of toxins are mainly responsible for the local tissue damage that may develop in severe envenoming by *Bothrops* species (3).

Currently, there are few countries in Latin America that produce antivenoms. In Peru, the National Institute of Health produces a polyvalent antivenom in equines, which is the only effective treatment against snake bites. The antivenom is obtained after successive subcutaneous injection of equines every 8 days with the venoms of several snake species. Blood is collected after a period of 3 months and plasma is processed to obtain the IgG fraction.

It has been calculated that probably less than 30% of the total antibodies from an equine antivenom are effectively neutralizing the toxins of the venom (22). Moreover, among important potential adverse effects of the antivenoms produced in equines are vasculitis, arthritis, and renal failure, which may originate from the formation of immunocomplexes between antivenom antibodies and the anti-horse antibodies that accumulate in blood vessels, joints, and glomeruli (22).

There is an urgent need to reduce the costs and to increase the efficiency of current antivenoms. In this context, many researchers have proposed novel alternatives to the common use of equine antibodies, such as recombinant antibodies from human or camelid VHH (23).

In this study, we have constructed a camelid (*Lama glama*) immune VHH library to retrieve Nbs against *B. atrox* venom using a phage display strategy. Several Nbs directed against hemorrhagic and myotoxic components were cloned and recombinantly expressed in *E. coli*. Their ability to neutralize hemorrhage and myotoxicity was screened in preincubation-type assays in mice, to reveal their potential as eventual therapeutic agents against snakebite envenoming, particularly against the local tissue pathology induced by venom SVMPs and PLA_2_.

## Materials and Methods

### Ethics Statement

Experiments involving animals were carried out in accordance with recommendations of the National Council for the Control of Animal Experimentation (CONCEA) and were approved by the Ethics Committee on Animal Use from the National Institute of Health from Perú under protocol IE003.

All mice were euthanized after experiments by CO_2_ inhalation. A llama was immunized with sublethal doses of snake venom according to previously reported doses for these animals, and their health statuses were continuously monitored by a veterinarian experienced in camelid management.

### Partial Purification of Hemorrhagic and Myotoxic Proteins of *B. atrox* Venom and *in vivo* Activity Assays

The venom of *B. atrox* was obtained from many individual snakes of the Peruvian region of Iquitos-Loreto. Venom was pooled, lyophilized, and stored at −80°C. A sample of 500 mg was dissolved in 2 ml of 10 mM HEPES buffer, pH 7.5, as this solution is accepted to keep the proteins in an active conformation. The solubilized venom was applied to a Sephadex G100 column previously equilibrated with the same buffer. Fractions of 2 mL/tube were collected at a flow rate of 12 mL/h. The protein peaks were monitored at 280 nm, collected, analyzed by SDS-PAGE, and tested for toxic activities.

The hemorrhagic activity was determined by intradermal injection in the ventral skin of mice (24), as described previously (25). The myotoxic activity was evaluated through the release of Creatine kinase (CK) to plasma (26). CK activity was determined using a commercial kit CK-NAC^∗^FS (Diasys) and expressed as the “minimum myotoxic dose” (MMD) (27). Tissue samples from the mice were obtained for histological evaluation.

### Immunization of *Lama glama* and Immune Response Monitoring

**FIGURE 1:**
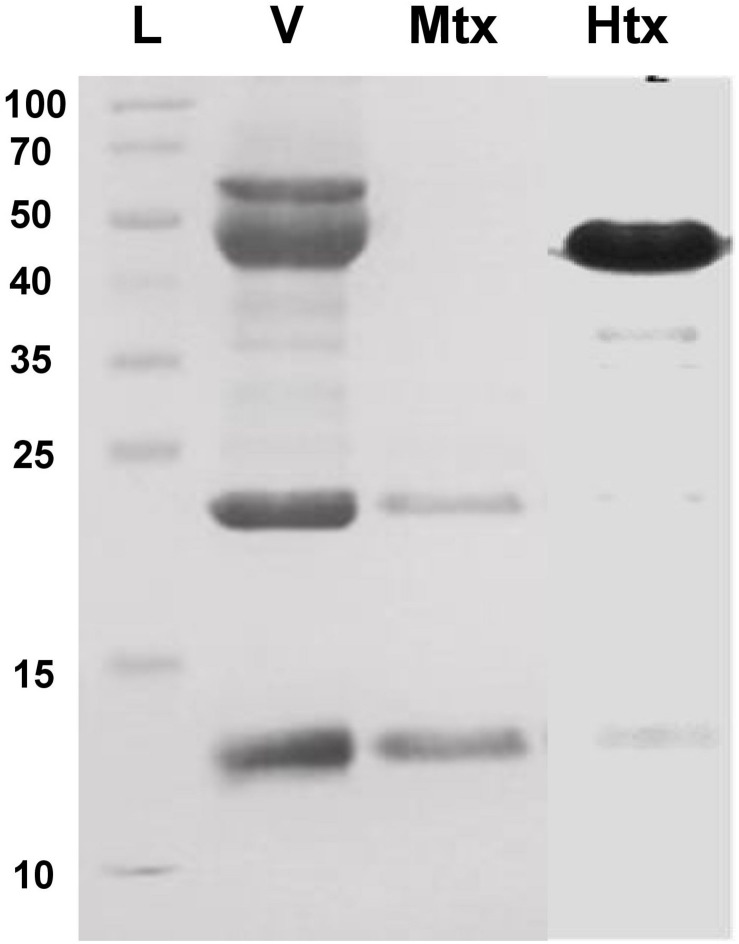
Immune response of llama against *B. atrox* snake venom and neutralization potency of hyperimmune serum. The venom protein-specific antibodies within the sera (diluted 3200 times) was evaluated using 50 ng of total *B. atrox* venom in an ELISA (black line). The open upward pointing arrows show the days that the llama was immunized and the black downward pointing arrow shows the time of bleeding to generate the Nb library. The neutralization potency of these serum antibodies against the lethal effect of the venom was also determined in mice (red line).

A two-year-old adult male *L. glama*, provided with food and water *ad libitum*, was immunized at weekly intervals for 10 weeks with increasing doses of *B. atrox* venom (0.5, 1, 2, 3, and 6 × 4 mg), mixed with Gerbu adjuvant (Gerbu Biotechnic), at a 1:1 ratio in 2 mL total volume of the inoculum. The mixture of venom and adjuvant was administered via subcutaneous injections in four nearby sites of the loin of the llama. After a waiting period, several additional injections were administered, as indicated in Figure 1.

The immune response of the llama was monitored by an enzyme linked immunosorbent assay (ELISA). Briefly, microtiter plates (Thermo Fisher Scientific) were coated with 50 ng of *B. atrox* whole venom diluted in 15 mM Na_2_CO_3_ and 35 mM NaHCO_3_, and incubated overnight at 4°C. Wells were washed with washing buffer (PBS; 0.05% Tween-20), and residual protein binding sites on the plastic were blocked with blocking solution (either 1% BSA or 2% skim milk in PBS) for 1 h at room temperature. Then, serial dilutions (1:50; 1:100;1:200; 1:400; 1:800; 1:1600, and 1:3200) of the llama serum, collected each week, as well as pre-immune serum, were added to the wells and the plates were incubated for 1 h at room temperature. The plate was washed five times, and goat anti-llama IgG-peroxidase conjugate (Thermo Fisher Scientific) at a 1:12000 dilution in blocking solution was added to each well and incubated for 1 h. The wells were washed again and finally, 100 μL TMB/H_2_O_2_ substrate (Invitrogen) was added to the wells. After 5–15 min incubation at room temperature and shielded from light, we added 50 μL of 0.5 M H_2_SO_4_ to stop the color reaction and absorbances were measured at 450 nm in a microplate reader (Multiskan Go, Thermo Fisher Scientific). Each sample was run in triplicate, and the pre-immune serum was used as negative control.

### Nanobody Library Construction

Llama whole blood (150 mL) was collected by venipuncture 3 days after the last immunization. Lymphocytes were isolated by centrifugation over a gradient of Ficoll-Paque Plus (GE Healthcare). Total RNA from the lymphocytes was extracted with Trizol Reagent (Invitrogen), purified by RNA Mini kit (Qiagen). The cDNA was synthesized by Superscript III Reverse Transcriptase (Invitrogen), and the first PCR with CALL001 and CALL002 primers was carried out. PCR products of around 700 and 900 bp were obtained, and the band of 700 bp was purified from agarose gel. This PCR product was used as the template for a second, nested PCR with specific primers carrying a recognition sequence for *Sap*I restriction enzyme at both 5′ ends, amplifying a product of about 500 bp corresponding to the VHH gene, which was purified by phenol/chloroform. The Restriction/Ligation reaction was set up in a single tube containing 1 μg of the pMECS-GG phagemid vector, 3 μg of VHH PCR product, 250 U of *Sap*I enzyme, 2.5 μl T4 DNA Ligase (15 U), 5 μl of 10 mM ATP, and 10 μl of reaction buffer, in a final volume of 100 μl. The pMECS vector encodes an influenza virus Haemagglutinin tag (HA) used for immunoassay selection and a 6x-His tag used for affinity purification of the expressed nanobody.

### Enrichment of Target-Specific VHH by Phage Display Biopanning

A representative aliquot of the Nb library was cultured in 330 mL 2xTY medium (supplemented with 100 μg/mL ampicillin, 2% glucose) at 37°C until the exponential growth phase was reached, infected with M13 helper phages, and incubated overnight at 37°C with continuous shaking. The bacteria were pelleted by centrifugation, and phage particles were recovered from the supernatant by precipitation with a concentrated polyethylene glycol solution (20% PEG and 2.5 M NaCl). Precipitated phage particles were resuspended in 1 mL PBS and quantified by spectrophotometry.

Two partially purified antigens of *B. atrox* venom with different enzymatic activities were used separately to select specific Nbs. A well of an ELISA plate was coated with antigen (15, 10, and 5 μg in the first, second, and third round of biopanning, respectively) in 0.1 M NaHCO_3_ buffer, pH 8.2. The plate was incubated overnight at 4°C, and residual protein binding sites were blocked with 2% skim milk for 2 h at room temperature. Then, 1 × 10^11^ phage particles were added and incubated for 30 min without shaking. After few washings, antigen-binding phages were eluted with 100 μl of freshly prepared triethylamine 100 mM, incubated for only 5 min at room temperature, and neutralized with 1.0 M Tris–HCl (pH 7.4). The phage particles were used to infect a fresh *E.coli* culture and used to initiate the next round of panning or spread on Petri dishes for titration.

### Selection of Clones, Expression, and Purification of Recombinant Nanobodies

Individual colonies were grown after the first or second round of panning, and the periplasmic extracts were obtained for the identification of clones with antigen-specific Nbs. An ELISA was carried out by coating with 2 μg/mL of antigen used in each biopanning (for each clone we foresee a well coated with and a well without the target protein). Incubation was carried out overnight at 4°C. After removal of excess antigen and washing the wells thee times with washing buffer (0.05% Tween 20 in PBS), we added 200 μl of blocking solution (2% BSA in PBS). Incubation was carried out for 2 h at room temperature. After removing the blocking solution and washing the wells five times with washing buffer, 100 μl of a 1/2 dilution in PBS of each periplasmic extract was added to the wells and incubated for 1 h at room temperature. Then, the cell extracts were removed, wells were washed five times with washing buffer, and 100 μl of a 1/2000 dilution of mouse primary antibody against the hemagglutinin tag was added and incubated for 1 h at room temperature. After washing the wells eight times, 100 μl of a 1/2000 dilution of anti-mouse IgG secondary antibody conjugated with Alkaline Phosphatase (Cell Signaling) was added. Incubation was carrued for 1 h at room temperature, followed by removal of unreacted antibody, eight washes with washing buffer, and, finally, the addition of 100 μl of the substrate solution for alkaline phosphatase. Absorbances were measured at 405 nm. The positive clones were identified when the absorbance value in wells with target protein was at least three times above the value of the signal in control wells lacking antigen. For all ELISA positive clones, we performed a colony PCR using the Nova Taq PCR Master Mix kit (Merck Millipore) to verify the insert size. Finally, the nucleotide sequencing of each clone was performed using the Applied Biosystems 3500 XL genetic analyzer.

Each ELISA positive clone (in TG1 cells) with a unique sequence was cultured, and a plasmid preparation was used to transform chemically competent WK6 *E. coli* cells. Recombinant WK6 clones were analyzed by colony PCR and DNA sequencing of their VHH gene to confirm that their DNA sequence was identical to that obtained for TG1 clones.

Unique clones were grown in 1 L of TB medium while shaking at 37°C to reach an OD at 600 nm of 0.6–0.9, before adding 1/100 volume 1 M IPTG and continue culturing for 14–16 h at 28°C. The bacteria were harvested by centrifugation, and cell pellets were resuspended in TES solution (Tris, EDTA, Sucrose). The tubes with the cell suspension were shaken at 200 rpm for 6 h at 4°C, then 2 volumes of TES/4 were added, and the shaking was continued overnight in the cold room. Finally, unique Nb extracts were purified on Ni^2^^+^ resin His60 Superflow. The captured his-tagged Nb was eluted from the Ni^2^^+^ resin with 500 mM imidazole in PBS. The Nb eluate was depleted of imidazole by diluting and concentrating by centrifugal ultrafiltration. The concentration of the purified Nb protein was determined by the Bradford method.

### Neutralization of the Hemorrhagic and Myotoxic Activity by Nanobodies

A first anti-hemorrhagic screening assay was performed by challenging groups of three mice intradermally with a quantity of the hemorrhagic protein (1.4 μg) known to produce sufficient hemorrhagic activity either alone or pre-incubated with 90 μg of each purified Nb for 1 h at 37°C.

In a second neutralization assay, the minimum hemorrhagic dose (MHD) of *B. atrox* whole venom was determined, and a challenge of 5 MHD of venom alone, or preincubated with the different Nbs, was tested in mice by intradermal injection. Free image analysis software (Inkscape 0.91) was used to determine the size of the area and intensity of hemorrhagic lesion (28).

For the neutralization test of myotoxic activity, 3 MMD (minimum myotoxic dose) was used as challenge (13.5 μg), in groups of six mice. The venom was preincubated with 40–70 μg of each purified Nb for 1 h at 37°C, and then 100 μl of the mixture was injected intramuscularly. Control mice received the same dose of venom preincubated with PBS, or a PBS injection alone. After 3 h, a blood sample was obtained from mice and the Creatine Kinase (CK) activity in plasma was determined.

### Neutralization of the Lethal Effect of *B. atrox* Venom in Mice by a Mixture of Nanobodies

Neutralization of the lethal effect of the venom was tested with a challenge dose of 4 LD_50_ (median lethal dose), equivalent to 16 μg intraperitoneally injected venom (LD_50_ = 3.96 μg). The venom was preincubated with a pool of 100 μg of each of four selected Nbs (H6, H8, H9, and M85) for 1 h at 37°C, and then 0.5 mL of the mixture was injected intraperitoneally in groups of six mice. Control mice received PBS alone or venom preincubated with PBS. The number of death mice was scored over a total time of 48 h. At the end of this 48 h, all dead and living mice were examined for hemorrhage of the peritoneal cavity.

## Results

### Partial Purification of Hemorrhagic and Myotoxic Proteins of *B. atrox* Venom and *in vivo* Activity Assays

**FIGURE 2:**
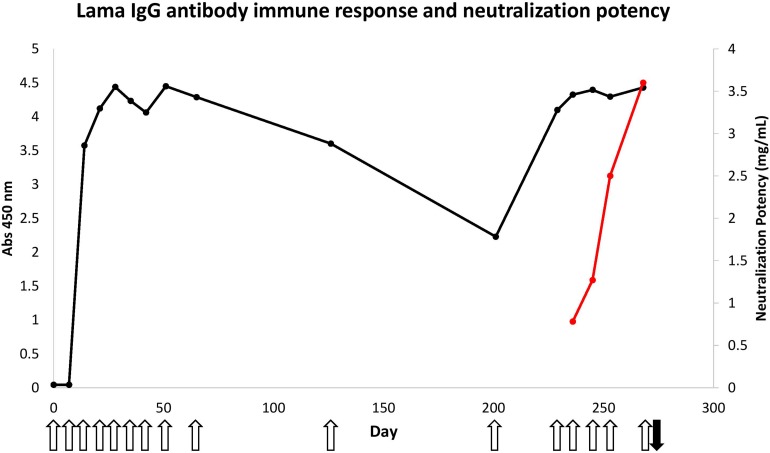
SDS-PAGE (15% acrylamide) of the hemorrhagic and myotoxic fractions from *Bothrops atrox* snake venom. L, protein ladder (Thermo Fisher Scientific #26623) with molecular masses in kDa at the left-hand side of the figure. V, *B. atrox* whole venom; Mtx: fraction with myotoxic activity; Htx, fraction with hemorrhagic activity.

*Bothrops atrox* venom proteins were fractionated by size exclusion chromatography and tested for hemorrhagic or myotoxic activity in mice. Hemorrhagic activity was detected in fractions 11–14, with fractions 13 and 14 showing 1.213 and 1.115 units (mm^2^/μg), calculated as the area of the hemorrhagic lesion divided by the protein concentration of the toxin. Of these, fraction 13 was selected for further assays because of its higher hemorrhagic activity. It showed a predominant protein band of 50 kDa after SDS-PAGE (Figure 2).

On the other hand, a high myotoxic activity was detected in fraction 18, showing two protein bands of 13 kDa and 23 kDa after SDS-PAGE (Figure 2), which correspond to the monomeric and dimeric forms of a phospholipase A_2_-like (Lys49) myotoxin. This was inferred by observing that the myotoxic fraction showed no detectable phospholipase activity on egg yolk phospholipids, nor anticoagulant effect on plasma, when up to 100 μg were assayed.

The intramuscular injection of aliquots of fraction 18 induced a clear myotoxic effect *in vivo*, evidenced by the increase of Creatine kinase activity in the plasma of treated mice, reaching a maximum at 3 h and returning to normal levels after 12 h. The minimal myotoxic dose (MMD) of this protein was calculated to be 4.5 μg. It induced a CK activity of 1891.7 U/L, compared to 192.1 U/L for plasma of a PBS-injected mice. Histological examination of the injected gastrocnemius muscle showed necrosis, edema, and inflammatory infiltrate.

### Llama Immune Response and Neutralization of *B. atrox* Whole Snake Venom by the Immune Serum

Immunization of a llama with *B. atrox* venom initially consisted of only nine doses. Enzyme-immunoassay monitoring of sera taken before each injection showed a rapid increase of the total amount of venom-specific antibodies in the serum (Figure 1). Three injections at weekly intervals were sufficient to reach a plateau signal for the antigen-specific antibodies within the serum diluted 3200 times, using only 50 ng of total venom containing multiple proteins at various concentrations.

However, despite this rapid antibody response, the neutralization potency of the immune serum against a lethal dose of *B. atrox* venom remained rather poor (0.78 mg/mL) after nine injections of venom, compared to the minimum required neutralization potency of Peruvian anti-bothropic antivenom produced in equines (2.5 mg/mL).

Therefore, it was decided to perform additional immunizations in an attempt to increase the neutralization potency of the llama serum. This appeared to be successful as the neutralization potency increased to 3.6 mg/mL, a level even higher than that obtained with equine antivenom (2.5 mg/mL) (Figure 2).

### Construction of a Llama Immune VHH Library

After immunization of the llama with *B. atrox* venom, we isolated about 5 × 10^6^ lymphocytes from peripheral blood, to extract total RNA that was reverse transcribed into cDNA, and used as template to amplify the VHH genes in a two-step nested PCR. The final VHH PCR amplicon was successfully ligated in pMECS-GG phage display vector and transformed in TG1 *E. coli* cells. We obtained a library of 1.78 × 10^9^ clones that was used for selection of Nbs.

### Phage Display Selection of Recombinant Nanobodies Against Hemorrhagic and Myotoxic Proteins

A total of 94 clones were picked, separately, after the first and second round of biopanning for screening against the two antigen fractions by ELISA. Eighty clones expressed a Nb that recognized the hemorrhagic protein, whereas 76 clones recognized the myotoxin.

Colony PCR with VHH specific primers was performed on all clones that scored positive in ELISA, and the DNA amplicons were sequenced by the Sanger method and translated to amino acid sequences. As expected, the VHH sequences showed a high degree of redundancy, especially among the clones from the second round of panning.

**FIGURE 3:**
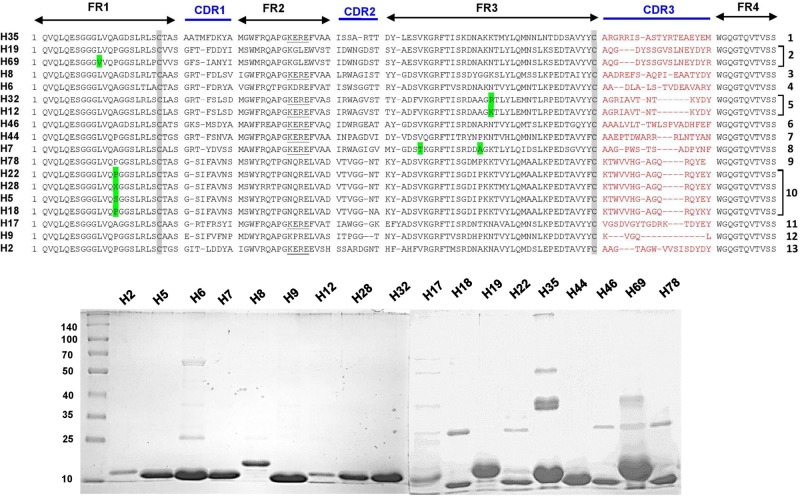
Amino acid sequences and SDS-PAGE of selected nanobodies against the hemorrhagic fraction of *B. atrox* venom. Upper panel: Selected nanobody sequences were grouped by CDR3. Framework Regions (FR); Complementarity-determining regions (CDR1, CDR2, and CDR3); amino acids highlighted in green are some variations in the consensus FR sequences. Lower panel: SDS-PAGE (10% acrylamide) of selected nanobodies. Molecular mass markers (in kDa) are shown in the first lane.

**FIGURE 4:**
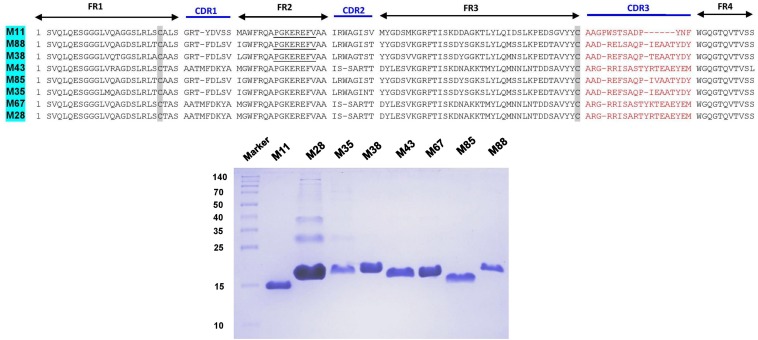
Amino acid sequences and SDS-PAGE of selected nanobodies against the myotoxic fraction of *B. atrox* venom. Selected nanobody sequences (top) were grouped by CDR3. Framework Regions (FR); Complementarity-determining regions (CDR1, CDR2, and CDR3). SDS-PAGE (15% acrylamide) of selected nanobodies (bottom). Molecular mass markers (in kDa) are shown in the first lane.

The complementarity determining regions (CDRs) and framework regions (FRs) of VHH were identified for 18 and 8 unique Nb clones against the hemorrhagic and myotoxic proteins, respectively. Based on their CDR3 sequences, these sequences were grouped into 13 clusters or families for the hemorrhagic protein and eight clusters for the myotoxic protein (Figures 3, 4). Differences among clones from the same family were located in FRs and CDRs as well.

### Expression and Purification of VHH

The plasmid DNA of one clone from each cluster, and against each of the two antigens, was transformed in WK6 *E. coli* cells and was cultured in 1 L in order to express the encoded Nb in the periplasm. The periplasmic protein extract was purified on nickel-ion containing resin, eluted with 500 mM imidazole and analyzed by SDS-PAGE.

The different nanobodies were obtained at variable yields, between 0.5 to 5 mg of purified recombinant nanobody per liter of culture. The vast majority of them revealed a single band corresponding to the MW predicted from its amino acid sequence (Figures 3, 4).

### Neutralization of Hemorrhagic Activity by Nanobodies

**FIGURE 5:**
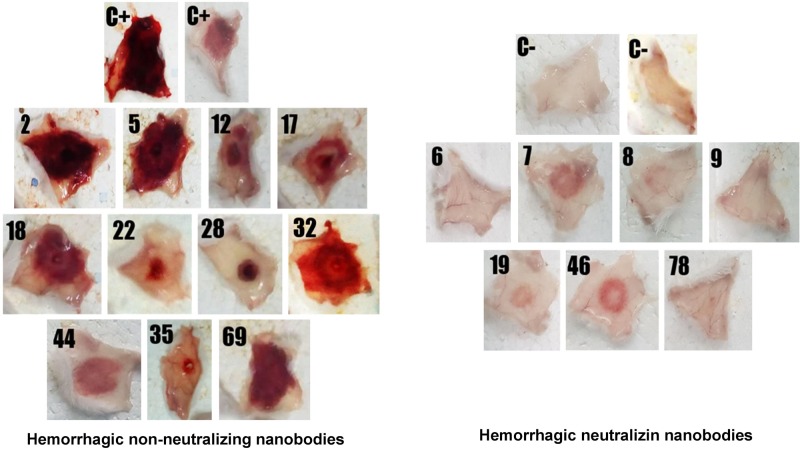
Screening of individual nanobodies against a hemorrhagic fraction of *B. atrox* venom to neutralize hemorrhagic activity in mice. Each nanobody (90 μg) was mixed with 1.4 μg of the hemorrhagic protein and incubated for 1 h at 37°C. Then, 100 μL of the mixture was injected intradermally in mice. After 3 h, mice were euthanized, and the internal hemorrhagic areas of the skin were recorded. C+, mice injected with hemorrhagic fraction alone; C–, mice injected with PBS. The group of nanobodies shown at the left did not neutralize hemorrhage, while the group at the right did.

A first screening assay revealed five hemorrhage-neutralizing Nbs (named H6, H7, H8, H9, H19, H46, and H78) which completely inhibited the hemorrhagic activity of the venom fraction, whilst some other Nbs neutralized it to a lower degree. When Nbs were tested against the whole venom, three of them (H6, H8, and H9) were able to fully neutralize the hemorrhagic activity (Figure 5).

The neutralization potency of the Nbs was concentration-dependent.

**FIGURE 6:**
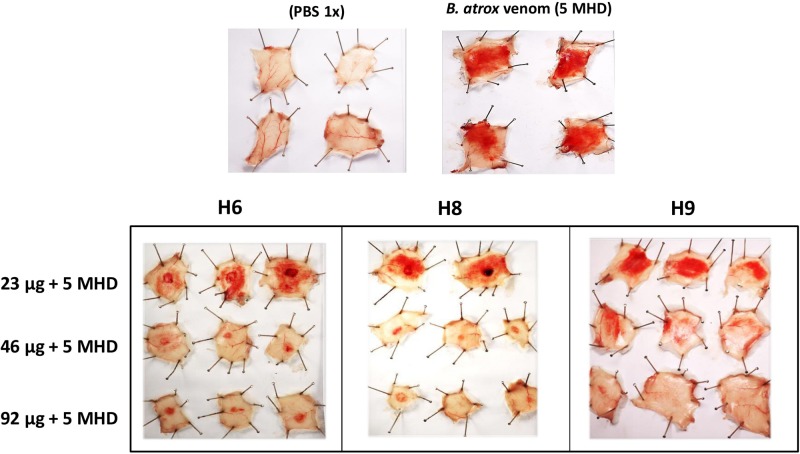
Neutralization of the hemorrhagic effect of whole *B. atrox* venom by nanobodies. Each nanobody (amounts indicated in μg in the legend at the left) was mixed with 5 fold minimum hemorrhagic dose (5 MHD) of venom and incubated for 1 h at 37°C. Then, 100 μL of the mixture was injected intradermally in mice. After 3 h, mice were euthanized, and the internal hemorrhagic areas of the skin were recorded.

Selected Nbs neutralized the hemorrhagic activity with some of them exhibiting somewhat different patterns of inhibition. For example, Nbs H6 and H8 left a few localized hemorrhagic spots even at the highest dose tested (92 μg Nb), whereas Nb H9 resulted in a diffuse pattern of residual hemorrhage, which disappeared with a dose of 92 μg Nb (Figure 6).

### Inhibition of Myotoxic Effect of Whole *B. atrox* Snake Venom

**FIGURE 7:**
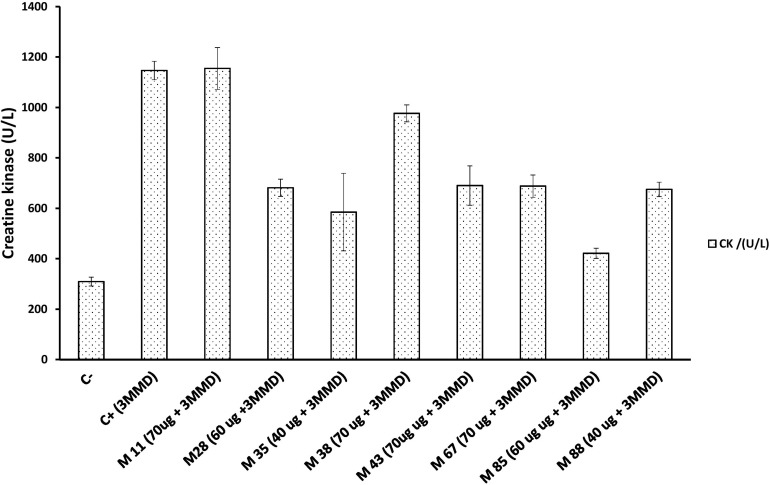
Neutralization activity of individual Nbs on the myotoxic effect induced by a *B. atrox* venom fraction. Each Nb was mixed with 3 fold minimum myotoxic dose (3 MMD) of protein (13.5 μg) and incubated for 1 h at 37°C. Then, 100 μL of the mixture was injected intramuscularly in mice. After 3 h, the plasma Creatine kinase activity was determined. C– and C+ represent the values for mice injected with PBS or venom alone, respectively. Bars represent mean ± SD of four mice.

Six out of the eight myotoxin-specific Nbs were able to decrease myonecrosis induced by the whole venom, as evaluated by the CK activity levels in plasma. These Nbs were M28, M35, M43, M67, M85, and M88, which blocked 40 to 63% of the myotoxic activity of a 3 fold minimum myotoxic dose (3 MMD) challenge with whole venom (Figure 7).

### Neutralization of the Lethal Effect of *B. atrox* Venom in Mice by a Mixture of Nanobodies

**FIGURE 8:**
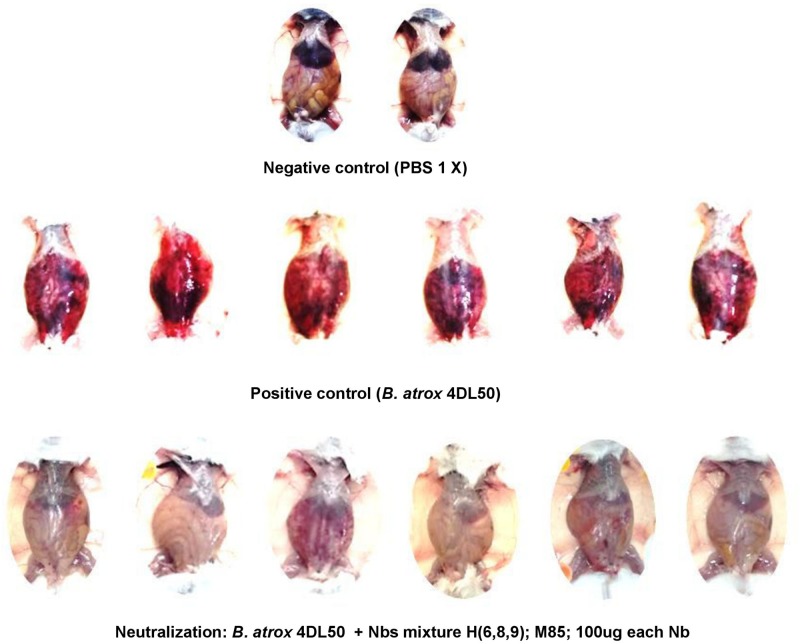
Neutralization of the hemorrhagic effect of whole *B. atrox* snake venom by a mixture of nanobodies. Venom alone (4 LD_50_) or preincubated with a mixture of nanobodies for 1 h at 37°C, was injected intraperitoneally in mice. Lethality was not prevented, but the widespread hemorrhage induced by the venom was completely inhibited by the nanobody mixture.

Nanobodies (H6, H8, H9, and M85) that neutralized the hemorrhagic and myotoxic activities of the venom fractions were mixed and tested for the possible neutralization of the lethal effect of the whole venom. However, this Nb cocktail was unable to prevent lethality. Nevertheless, an examination of the peritoneal cavity of the animals revealed that the widespread hemorrhage caused by the venom in the control group was effectively prevented by the Nb mixture (Figure 8).

## Discussion

Two main tissue-damaging components of *B. atrox* venom, with hemorrhagic and myotoxic effects, respectively, were used as antigens to select a set of Nbs from an immune llama library by a phage-display enrichment. The hemorrhagic fraction obtained likely corresponds to a PIII metalloproteinase (SVMP III) reported to be the most hemorrhagic toxin of this venom, with a molecular mass of 50 kDa, in agreement with the main band observed in SDS-PAGE. Moreover, the hemorrhagic activity analysis of *B. atrox* venom chromatographic fractions showed two peaks, consistent with the presence of two distinct metalloproteases (17), a highly active SVMP-III and a weaker SVMP-I. These hemorrhagic proteins represented the most abundant proteins in a proteomic study on the venom composition, accounting for nearly 70% of the total protein contents of the *B. atrox* venom (17).

On the other hand, the myotoxic protein fraction showed biochemical and functional features that are consistent with those of the previously reported Myotoxin I of *B. atrox*, a PLA_2_ homolog of the Lys49 type (29), devoid of catalytic activity. This type of PLA_2_-like myotoxin is highly conserved within the genus *Bothrops*, and found in many crotalid snake venoms of both Old and New World species (30).

In order to obtain Nbs against relevant tissue-damaging components of *B. atrox* venom, a llama was immunized following an initial scheme of nine injections at weekly intervals. In spite of the positive serum antibody response, neutralization potency remained low, and therefore immunization was continued with seven additional boosts with the venom. This resulted in an increase of the venom neutralization potency of the anti-serum, conveniently explained by the affinity maturation of the antibodies for their cognate antigens, compared to the first, rapidly raised antibody population.

From the pool of Nb genes of the immunized llama, a set of Nbs were obtained against the hemorrhagic or myotoxic venom fractions, respectively. The Nbs showed great diversity, evidenced by the number of different sequences obtained after one and two rounds of panning. These nanobodies were grouped into several clusters based on sequence identity in their CDR3, the region of VHH with the largest variability.

Selected clones from each cluster were expressed in *E. coli* and purified from cultures at a 1 L scale, with variable yields. SDS-PAGE analysis of these Nbs showed a slight variation in molecular weight that was in agreement with the Nb nucleotide sequences and translation into amino acid sequences.

We then searched for Nbs capable of neutralizing two of the most important pathological effects of *B. atrox* venom, namely hemorrhage and myotoxicity. From the 18 and 8 Nbs obtained against the hemorrhagic and myotoxic fractions, respectively, several displayed neutralizing activity.

We identified Nbs H6, H8, and H9 to possess the best neutralizing activity for the hemorrhagic effect of the snake venom, and Nbs M28, M35, M43, M67, M85, and M88 as good inhibitors of the myotoxic effect, albeit with some variations in their neutralizing capacity.

It is important to consider that Nb clones of very similar sequence, even in their CDR3 region, but having one or more mutations in other regions such as CDR1 or CDR2, may have different neutralization capacity due to differences in affinity for their target. Interestingly, one of the Nbs with the shortest CDR3 region (H9) was the best neutralizer of the hemorrhagic activity of the whole venom.

Three of the Nbs were able to neutralize the hemorrhagic activity of the whole venom, suggesting that they should inhibit the activity of both the high molecular weight SVMP-III toxins and the smaller SVMP-I. An alternative interpretation is that the SVMP-III component plays a more predominant role in the hemorrhagic effect of the whole venom, in comparison to the SVMP-I, generally known to be of weaker activity (31). Addressing the fine specificity of the toxins and epitopes recognized by these Nbs, in future studies, will shed light on the detailed mechanisms of neutralization.

Some of the obtained Nbs neutralized effectively local pathological effects of venom toxins, such as bleeding and muscle damage, and a mixture of them prevented the hemorrhagic action of the whole venom. However, the neutralizing Nbs were unable to prevent the lethal effect of the venom. This is an interesting observation, which suggests that the lethal action of the venom in this mouse model most likely involves the participation of additional toxins, for example, enzymes and non-enzymatic proteins that may affect hemostasis causing thrombosis, platelet alterations, or inducing acute kidney damage and hypotensive actions (32). Further studies should focus on the identification of Nbs against additional components of *B. atrox* venom having systemic toxicity involved in the lethal effect.

In this study, we have tested the neutralizing ability of Nbs in preincubation-type assays, as a first indicator of Nbs for their potential as future therapeutic agents against snakebites. Nbs to snake venoms have been obtained with this purpose in previous studies (33, 34), and there are wide possibilities of engineering a variety of molecular formats based on these antigen-specific structures (22, 35–37). Future work on the Nbs described here will address their preclinical neutralizing performance when administered after envenoming, as well as their potential for enhancing the neutralizing potency of current therapeutic antivenoms against snake venom-induced local tissue damage.

## Data Availability Statement

The raw data supporting the conclusions of this article will be made available by the authors, without undue reservation, to any qualified researcher.

## Ethics Statement

The animal study was reviewed and approved by the Institutional Committee of Ethics and Research, Instituto Nacional de Salud, Peru.

## Author Contributions

HB led the research team. CB contributed to the snake venom collection and first analysis. BT, JL, and EC contributed to the llama veterinary management, immunizations, and blood sample collections. VY, WL, OC, CP, MG, DG, HM, MC, GR, ER, and NS contributed to the experiments of library construction, nanobody selections, and functional experiments for nanobodies. HB, VY, WL, OC, CP, MG, CV, SM, and BL contributed to the data analysis. HB, VY, SM, and BL contributed by writing the manuscript. All authors revised and agreed with the submission of the manuscript.

## Conflict of Interest

The authors declare that the research was conducted in the absence of any commercial or financial relationships that could be construed as a potential conflict of interest.
